# Assessing mental well-being in a Sinhala speaking Sri Lankan population: validation of the WHO-5 well-being index

**DOI:** 10.1186/s12955-020-01532-8

**Published:** 2020-09-11

**Authors:** B. P. R. Perera, R. Jayasuriya, A. Caldera, A. R. Wickremasinghe

**Affiliations:** 1 Department of Public Health, Faculty of Medicine, University of Kelaniya, P.O. Box 6, Thalagolla Road, Ragama, 11010 Sri Lanka; 2 School of Public Health & Community Medicine, University of New South Wales, Randwick, NSW 2052 Australia

**Keywords:** WHO-5 Sinhala version, Validation, Well-being, Sri Lanka

## Abstract

**Purpose:**

The WHO-5 well-being index is a widely used, short rating scale that measures subjective well-being. We translated the WHO-5 index into Sinhala and tested its psychometric properties including measurement invariance among diverse groups in a community sample in Sri Lanka.

**Methods:**

The sample of 267 persons aged between 16 and 75 years was recruited from a semi-urban area. 219 completed a paper-based questionnaire and 48 responded to an online survey. Construct validity was tested for factorial validity (Confirmatory Factor Analysis -CFA), convergent validity and known group validity. Composite reliability for congeneric measures and test-retest reliability were also tested. Multi-group CFA (MG-CFA) was used to test measurement invariance.

**Results:**

The translated Sinhala version demonstrated good content and face validity. Internal consistency reliability of the five items had a Cronbach’s alpha of 0.85 and test-retest reliability over 2 weeks was satisfactory (Pearson *r* = 0.72, *p* < 0.001, ICC = 0.82). Confirmatory factor analysis supported factorial validity with a ${\chi}_5^2$ =4.99 (*p* = 0.28), a RMSEA of 0.03 (90% C.I. =0.00–0.10), a SRMR of 0.02, a TLI of 0.99 and a CFI of 0.99; factor loadings were between 0.55 and 0.89. Measurement invariance was acceptable for configural, metric and scalar invariance for gender.

WHO-5 scores were significantly negatively correlated with the Patient Health Questionnaire (PHQ-9) (Pearson’s *r* = − 0.45, *p* < 0.001) scores and the Kessler Psychological Distress Scale (K10) scores (Pearson’s *r* = − 0.56, *p* < 0.001).

**Conclusion:**

The Sinhala translation of WHO-5 well-being index has shown acceptable psychometric properties and can be used for assessing mental well-being in the community in Sri Lanka. Further testing of the measure with larger and diverse (including different ethnic/cultural) groups are indicated to test measurement invariance of the measure.

## Introduction

The World Health Organization-5 (WHO-5) well-being index was developed in response to the need of an instrument to measure subjective well-being which reflects a single dimension with high clinical face validity [1]. WHO-5 was derived from WHO-10 which has its origin from a 28-item rating scale that was used in a multi-centre study in eight European countries [2]. The 10 items of the WHO-10 were selected from the 28-item scale using item response theory (IRT) analysis [2]. WHO-10 includes items phrased positively reflecting well-being and phrased negatively to reflect distress [2]. As the WHO definition of good health considers positive well-being reflecting mental health, the WHO-5 comprises only the positively phrased items of WHO − 10.

A systematic review of its use found that it has been translated into more than 30 languages and has been used in research studies globally [1, 3]. The validity of the WHO-5 has been tested in different countries and settings, but predominantly in high income settings in the west and Japan [4–14] as a robust outcome measure of well-being [4, 6, 8, 9, 15, 16]. However, there are only a very few validations in Low- and Middle-Income Countries (LMIC), and specifically in Asia [15, 17]. The aim of this study was to assess the psychometric properties of a translation of the WHO-5 well-being index into Sinhala in Sri Lanka, a South Asian country.

In our review of the corpus of validation studies including the systematic review [1], only two studies have been conducted to test measurement invariance to assess whether the WHO-5 is being understood and interpreted in a similar manner by diverse groups such as by gender, age group, education levels and different culture/language groups in each setting [18, 19]. Therefore, the second aim of the study was to test measurement invariance of the WHO-5 among diverse groups using the Sinhala language.

## Methods

### Participants and recruitment

A community sample was recruited by contacting households in a sub-division of a district demarcated for population health services, called a Medical Officer of Health (MOH) area, in a semi-urban setting in the western province of Sri Lanka. Staff of the MOH obtained consent from Sinhala speaking participants 16 to 75 years of either sex for research assistants to visit on pre-arranged days and administer the questionnaire at their homes. In addition, an online version of the questionnaire was posted using Google Forms and accessible to participants who were contacted via word of mouth using a snowballing technique.

### Scales used

#### WHO-5 well-being index

The five statements in the WHO-5 well-being index are: 1) ‘I have felt cheerful and in good spirits’, 2) ‘I have felt calm and relaxed’, 3) ‘I have felt active and vigorous’, 4) ‘I woke up feeling fresh and rested’ and 5) ‘My daily life has been filled with things that interest me’. The degree to which these feelings were present in the last 14 days were scored on a 6 -point Likert-type scale ranging from 0 (“at no time”) to 5 (“all of the time”). The item scores were summated to obtain an individual score ranging from 0 to 25, with higher scores indicating higher levels of well-being. As scales measuring health-related quality of life are conventionally translated to a percentage score, the summated score was transformed to a 0–100 score by multiplying the summated score by 4 [3].

#### Translation and content validation of the WHO-5 well-being index to Sinhala

The scale was translated into Sinhala language following an accepted process [20]. The original scale was translated into Sinhala language by two independent bilingual translators. The translation was then synthesized into one version by an investigator. This synthesized version was then back translated into English by two independent bilingual translators who were not aware of the original scale. Discrepancies were resolved to obtain the final version of the translated scale. The process was done for several iterations until the back translation to English was similar to the original English version of the questionnaire. An expert committee consisting of two experts who are familiar with the construct of interest, a methodologist, and both the forward and backward translators was convened. Content and face validity of the Sinhala translation was assessed by the expert panel [21].

The WHO-5 instrument was subjected to a cognitive test with 10 respondents to assess clarity and appropriateness of the wording [22]. The respondents were asked to complete the questionnaire and then were asked to describe what they understood by each item and why they selected a particular response for that item to assess whether it matched with the meaning of the original item.

#### Patient health questionnaire (PHQ-9)

The PHQ-9 depression module is a nine-item scale derived from the full PHQ. The severity of the symptoms is rated on a scale from 0 to 3 which corresponds to “not at all” to “nearly every day”, respectively. The PHQ-9 score can range from 0 to 27, higher values indicating increasing severity [23]. The translated questionnaire was tested among a Sinhala speaking Sri Lankan population and found to have good reliability with a Cronbach’s alpha of 0.90 and convergent validity [22].

#### Kessler psychological distress scale (K10)

The Kessler psychological distress scale is a mental health screening tool, used in population surveys, that is based on a 10-item five-point Likert type scale ranging from 0 to 4; it assesses the level of anxiety and depressive symptoms a person may have experienced in the past 4 weeks [24]. The translated questionnaire has been tested for its criterion validity using SCID in a Sinhala speaking population in Sri Lanka [24].

### Sample size

There are several factors that affect the sample size requirement for structural equation model (SEM) testing which include the complexity of the model, number of parameters to estimate and reliability of measures [25]. Using one such heuristic, the N:q rule, given that there are 15 parameters to estimate in a congeneric model for Confirmatory Factor Analysis [26], a sample size of 150 (for a 10:1 ratio) and 300 (for a 20:1 ratio) can be considered minimal and good, respectively [25].

### Psychometric analysis

#### Reliability

Cronbach’s alpha was used to assess internal consistency reliability; an α > 0.7 and item-total correlation coefficients of > 0.5 were considered to indicate satisfactory reliability. Composite reliability for congeneric measures were tested using confirmatory factor analysis (CFA) for all standard factor loadings of 0.6 or above [25].

The test-retest reliability of the scale within a span of 2 weeks was assessed in a sample of 38 participants using the Pearson’s product-moment correlation coefficient and Intraclass Correlation Coefficient (ICC) based on the absolute agreement in a two-way mixed method model. Reliability was classified as poor (ICC < 0.5), moderate (ICC 0.5–0.75), good (ICC 0.75–0.9) or excellent (ICC > 0.90) [27].

#### Convergent and discriminant validity

Convergent validity was tested with the validated Sinhala versions of PHQ-9 and K10 using the Pearson correlation coefficient. We hypothesized a negative correlation between WHO-5 and K10 and PHQ-9. A correlation of <− 0.5 was considered to indicate satisfactory convergent validity [26, 28].

Known-group validity was used to assess discriminant validity. We hypothesised that there would be no difference by gender, ethnicity and religion. We expected to find a higher mean well-being score among older persons and those in higher education categories [29]. Those who scored higher in PHQ-9 and/or K10 indicating presence of depressive symptoms and/or anxiety were expected to have low scores in WHO-5.

#### Construct validity

CFA was used to test the factorial validity of the single factor congeneric model. As the response options were based on a six-item Likert scale, which had ordinal properties, best practice recommendations were followed to analyse the data using the mean and variance adjusted weighted least square (WLSMV) estimator which is also robust to non-normality of the data [30]. The CFA model testing procedures of model specification, identification, and estimation were followed to test model fit [25]. As the *χ*2 is known to overestimate minor model mis-specifications, CMIN/DF ($\frac{\chi^2}{df}$) was used to assess model fit and ratios not exceeding three was considered an acceptable fit [31]. Four other approximate fit indices were considered: RMSEA *<* 0.050 and < 0.080 for close and reasonable fit, respectively: Comparative fit index (CFI) and Tucker–Lewis Index (TLI) of *>* 0.900 and > 0.950 were considered for acceptable and excellent fit, respectively [32]; Standard Root Mean Square Residual (SRMR) of < 0.05 was also considered because this index is independent of the estimator used [33]. Since we used the DWLS estimator for our analysis, SRMR has been recommended as the main index to test model fit [34–37].

#### Measurement invariance testing

Testing measurement invariance can be considered under the wider concept of bias in construct validity which has been defined as “the extent to which a test measures different constructs for different groups” [38]. Bias is said to be present if a measure systematically overestimates or underestimates scores on a variable for members of a particular group [38]. Bias can manifest itself in many ways either at the item level (DIF) or at the scale level (differential test functioning, DTF) [39].

While there are many methods to test MI; Multi-Group CFA (MG-CFA) is a widely accepted approach, [40, 41] and is widely available in different statistical programmes (e.g., Mplus, R, EQS, etc.), and it can be conducted with ordinal data. In the context of ordinal/categorical item data, MG-CFA has been shown to be more sensitive than item response theory (IRT) in identifying items with DIF with a lower rate of false positives [42].

Invariance testing using MG-CFA involves examining the fit of a series of increasingly restrictive models [25, 39, 43]. The researcher begins by testing a baseline model with the groups and, if fit is established, proceeds by imposing increasingly restrictive equality constraints (see Kline 2016 for details). If model fit does not meaningfully change when equality constraints are included, then the measure is said to be equivalent/invariant [39].

Measurement invariance was assessed based on sex (male/female), age (above 40/below40), occupational status (working/not working) and method of data collection (paper-based/online).

### Data analysis

Descriptive statistics are reported as frequencies and percentages or as means (±SD). Differences of means between groups were tested using independent sample t-tests or analysis of variance (ANOVA) and Student Neuman Keuls test for post-hoc comparisons. Statistical analysis was performed using IBM SPSS software version 22, CFA using lavaan 0.6–7.1565 and semPlot [44] packages in R software. Measurement invariance testing was conducted using Mplus 7.2.

### Ethics considerations

Ethics approval was obtained from the Ethics Review Committee of the Faculty of Medicine, University of Kelaniya, Sri Lanka (Ref.No.P/128/06/2019). Informed consent was obtained from the participants in writing prior to responding to the questionnaire and the scales.

## Results

### Sample characteristics

Data were collected from a community sample and an online sample. In the community sample, 221 paper-based questionnaires were distributed, of which, 197 were returned (response rate 89%). Eleven questionnaires were incomplete and were not used in the analysis. Seventy persons completed the online version of the questionnaire. In the final sample of 267 participants, the mean age was 34.1 years (SD 13.5 years; range 17 to 73 years), 72% were females and 96% were Sinhalese (4% were Tamils and Muslims who were conversant in Sinhala). The majority were Buddhists (71%), 42% of the sample had received an education up to grade 13, 58% had a pursued higher education, 45% were employed, 23% were unemployed and 32% were students (Additional file 1).

### Psychological well-being of the study participants

The mean (±SD) score of the WHO-5 index was 56 (±23.9). The mean (±SD) of the PHQ-9 score was 6.0 (±4.9) with 58 persons (22%) having a PHQ-9 score ≥ 10 indicating having depressive symptoms. The mean (±SD) score of K10 was 10.1 ± 7.5, ranging from 0 to 40: using a cut-off value of 16, 59 (22%) individuals had psychological distress.

### Psychometric properties of Sinhala version of WHO-5

#### Reliability

The Cronbach’s alpha for the Sinhala version of the WHO-5 index for the whole group was 0.854; and item-total correlations were above 0.5 (range 0.61 to 0.75). Composite reliability for congeneric measures as tested using CFA showed that all standard factor loadings were 0.5 or above, ranging from 0.5 to 0.88 (Table 1).

**Table 1:** Reliability of Sinhala version of WHO-5 well-being index

Item	Corrected item-total correlation	Squared multiple correlation	Cronbach’s alpha if item deleted	Factor loadings in CFA
I have felt cheerful and in good spirits	0.707	0.541	0.814	0.843
I have felt calm and relaxed	0.748	0.594	0.802	0.893
I have felt active and vigorous	0.635	0.427	0.832	0.725
I woke up feeling fresh and rested	0.640	0.421	0.831	0.557
My daily life has been filled with things that interest me	0.611	0.395	0.838	0.666

The Pearson correlation coefficient and the ICC used to assess test-retest reliability over 2 weeks were 0.72 (*p* < 0.001) and 0.82 (0.666–0.911) (*p* < 0.001), respectively, indicating high test-retest reliability over 2 weeks (Table 2).

**Table 2:** Intraclass correlation coefficient between initial test and re-test (*n* = 38) for the WHO-5 Sinhala version

Item	ICC	95% Confidence Interval	***p***-value
I have felt cheerful and in good spirits	0.707	0.443–0.847	< 0.001
I have felt calm and relaxed	0.749	0.521–0.869	< 0.001
I have felt active and vigorous	0.638	0.296–0.813	0.002
I woke up feeling fresh and rested	0.730	0.487–0.859	< 0.001
My daily life has been filled with things that interest me	0.738	0.502–0.863	< 0.001

#### Convergent and discriminant validity

Overall, there was a significant inverse relationship between WHO-5 and PHQ-9 scores for the full sample (Pearson’s *r* = (− 0.45), *p* < 0.001) and for both the paper-based (*r* = (− 0.46), *p* < 0.001) and online samples (*r* = (− 0.59), *p* < 0.001). The correlation between the Sinhala version of WHO-5 and K10 for the full sample (*r* = (− 0.56), *p* < 0.001) and for both paper-based (*r* = (− 0.56), *p* < 0.001) and online samples (*r* = (− 0.57), *p* < 0.001) were significantly negatively correlated indicating good convergent validity.

There was no difference between the mean WHO-5 scores by sex, ethnicity or religion (Table 3). The mean WHO-5 score of those with only a school education was significantly higher than that of those with higher education (t_265_ = 2.62, *p* < 0.05). The mean WHO-5 score of students was significantly lower than those employed and unemployed (F_2,264_ = 8.06, *p* < 0.05).

**Table 3:** Association between WHO-5 Sinhala version scores and selected variables

Sub group		WHO-5 (mean ± SD)	p-value
Age Category	age <=40 (*n* = 202)	55.00 ± 24.3	0.234^a^
	age > 40 (*n* = 65)	59.08 ± 22.5	
Sex	Male (*n* = 75)	52.68 ± 25.0	0.158^a^
	Female (*n* = 192)	57.28 ± 23.3	
Ethnicity	Sinhala (*n* = 256)	56.20 ± 23.9	0.504^a^
	Tamil and Moor (*n* = 11)	51.28 ± 24.3	
Religion	Buddhist (*n* = 189)	54.24 ± 24.4	0.059^a^
	Other (*n* = 78)	60.32 ± 22.2	
Educational Status	School education up to grade 13 (*n* = 112)	60.48 ± 23.5	0.009^a^
	Degree and above (*n* = 155)	52.76 ± 23.7	
Employment Status	Not employed (*n* = 62)	61.08 ± 23.1	0.001^b^
	Student (*n* = 84)	47.60 ± 23.8	
	Employed (*n* = 121)	59.20 ± 23.0	

^a^t-test ^b^ANOVA

#### Factorial validity

**Fig. 1:**
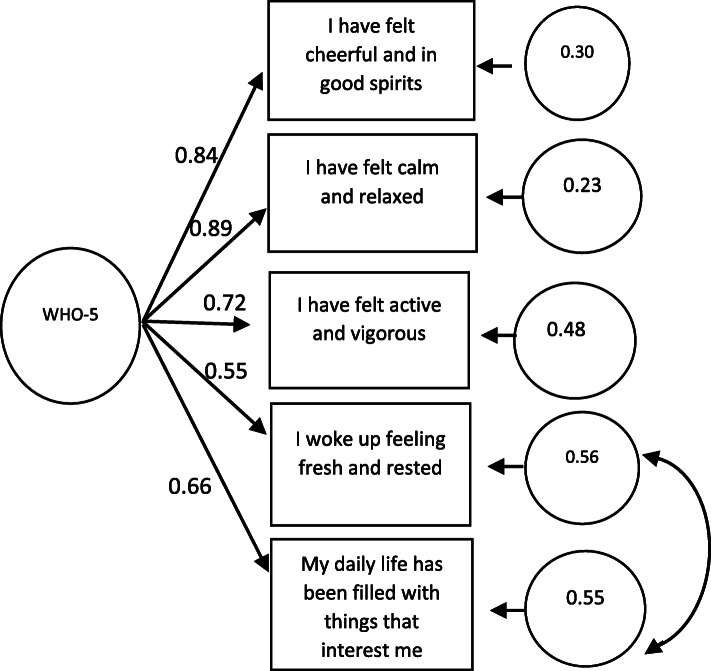
Path diagram of confirmatory factor analysis of a single factor model

The confirmatory factor analysis partially supported the one factor hypothesis with ${\chi}_5^2$ =22.934 (*p* = 0.0003), $\frac{\chi^2}{df}$ = 2.09, RMSEA of 0.116 (90% C.I. =0.071–0.166), a TLI of 0.98, a CFI of 0.99 and a SRMR of 0.03 (Fig. 1). Model fit indices recommended allowing covariances between items 4 and 5 which improved the model significantly; ${\chi}_5^2$ =4.99 (*p* = 0.28), $\frac{\chi^2}{df}$ = 1.89, RMSEA of 0.03 (90% C.I. =0.00–0.10), a TLI of 0.99, a CFI of 0.99 and a SRMR of 0.02.

Measurement invariance for gender is presented in Table 4.

**Table 4:** Fit statistics for measurement equivalence/invariance of WHO-5 scores across gender groups

				***∆χ***^**2**^								
Model	***χ***^**2**^	df	*p*	***∆χ***^**2**^	***∆DF***	*p*	CFI	***∆CFI***	TLI	RMSEA	RAMSEA CI	***∆RMSEA***
1.Configural invariance	27.38	21	0.15	–	–	–	0.997	–	0.997	0.048	0.000–0.093	–
2.Metric invariance	35.35	25	0.08	9.78	4	0.04	0.995	0.002	0.996	0.056	0.000–0.095	0.008
3.Scalar invariance	45.30	34	0.09	13.00	9	0.06	0.994	0.003	0.997	0.050	0.000–0.085	0.002
4.Strict invariance	55.24	39	0.04	14.74	5	0.01	0.992	0.005	0.996	0.056	0.010–0.088	0.008

Configural invariance for gender groups was established as the χ^2^ was acceptable (*p* > 0.05) and fit indices of Model 1 were within specified ranges (CFI = 0.997; RMSEA =0.048 (CI: 0.00–0.09)). Next a metric invariance model (Model 2) was tested. This model too had acceptable fit, the χ^2^ having a *p* > 0.05 with fit indices within specified ranges (CFI = 0.997; RMSEA =0.06 (CI: 0.00–0.095)).

The DIF test (χ^**2**^) was however significant (*p* < 0.05). As the fit indices (**∆**CFI < 0.01 and **∆**RMSEA < 0.015) were acceptable, it supported metric invariance. Subsequently, a scalar/threshold invariance model was tested (Model 3) where factor loading and thresholds were constrained to be equal. This model had adequate fit (χ^2^ was *p* > 0.05); CFI = 0.994; RMSEA = 0.05(CI: 0.00–0.085). The DIF test (*∆χ*^2^) was not significant (*p* > 0.05), and change in fit indices were acceptable (∆CFI < 0.01 and *∆* RMSEA < 0.015) supporting scalar invariance.

Finally, a strict invariance test (Model 4) was tested. This model had marginal fit (χ^2^ was *p* < 0.05); but CFI = 0.992; and RMSEA = 0.056 (CI: 0.10–0.088). The DIF test (*∆*χ^2^) was significant (*p* < 0.05), but the change in fit indices were acceptable (∆CFI < 0.01 and *∆* RMSEA < 0.015) providing mixed support for strict invariance.

Invariance tests for occupation, age and method of data collection were not supported as the baseline models were mis-specified [45]. Detailed results can be obtained from the researchers.

## Discussion

This study had its aim to assess psychometric properties of a translated version of the WHO-5 in Sinhala language, the main language used by the majority, in a community sample in Sri Lanka. Following accepted translation, the study provides evidence of good reliability and validity of the scale. We report a Cronbach’s alpha of 0.85 for the Sinhala translation which can be considered as very good even though the Persian and Taiwanese translations reported a higher Cronbach’s alpha (α = 0.94). Other measures of reliability such as test-retest reliability assessed on a sample of 38 and congeneric reliability add to the evidence that the Sinhala translation of WHO-5 is a reliable tool. However, the small sample size used to assess test-retest reliability may have an impact on the ICC at item level.

Factorial validity of the WHO-5 was tested using CFA. The chi-square test result often varies according to the sample size [25]. Both the CFI and TLI showed excellent fit, despite a high RMSEA. Values of RMSEA > 0.05 have been reported in other validation studies of WHO-5 [12, 13]. Reasons for similar misfit of RMSEA while other fit indices have been satisfactory have been reported in the literature [46]. Our study found that the residual variances for items 4 and 5 were borderline at the higher end. When tested in a post-hoc model covarying the two items, a non-significant Chi-square (*p* > 0.05) and better fit indices with RMSEA < 0.05 were obtained. Xia and Yang state that further studies are needed to seek alternative methods for goodness-of-fit evaluation with ordered categorical data [35]. Shi et al. has demonstrated that SRMR is relatively free from the choice of estimation method and the same population cut-offs can be applied regardless of the estimation method employed [34]. In the tested model a SRMR< 0.05 indicates acceptable model fit. Based on all of these considerations we feel there is sufficient support for factorial validity of the translated WHO-5. However, we recommend that further tests of validity be undertaken in larger and more generalizable samples.

We tested convergent validity of the WHO-5 comparing with two well tested measures of depression and anxiety that had been previously validated in Sri Lanka. In this population, the correlation between WHO-5 and PHQ-9 scores is at the lower end of the range (− 0.39 to − 0.73). Such values have also been found in other studies, [12, 13, 15, 47]. WHO-5 scores were negatively correlated with K10 scores.

The mean WHO-5 score for the Sinhala speaking Sri Lankan population with a mean age of 34 years was 14 (±5.9). This score is similar to the score of 14.3 (± 4.4) reported in a Thai validation study for a population with a mean age of 44 years [48]. We found no significant differences in the mean scores by age, gender, ethnicity or religion. The finding that the mean score was significantly lower in those with a higher level of education was contrary to our hypothesis. This is further explained by the low mean score among students, who were mostly from a University. University students are known to experience higher levels of distress due to academic pressure and burnout [49]. The unemployed group comprised mostly home makers with the majority being females. It has been reported that homemakers are as happy as those who work in salaried jobs [50].

The second aim of the study was to test measurement invariance of the WHO-5 among diverse groups using Sinhala language in Sri Lanka. We tested measurement invariance between groups by gender, age (categorised as <= 40 and > 40), occupation and method of administration. The results of measurement invariance by gender showed that the five items of the WHO-5 measure well-being in both males and females similarly for within group analyses, mean group comparisons, and selection purposes. The strict invariance test provided ambiguous findings but needs to be interpreted cautiously. There is yet debate about the use of this step in measurement invariance analysis [39]. Some authors have claimed that requiring residual invariance prior to latent mean comparisons is unrealistic and that lack of residual invariance may not meaningfully influence latent mean comparisons [40, 51, 52]. However, our examination of measurement invariance by age, occupation and method of administration encountered a mis-specified configural model and further testing was not possible [45].

The literature cautions that small samples (various authors give different values) are more prone to error in model fit indices in CFA and MG-CFA [53, 54]. As the groups by age, occupation and method of administration were unequal, minimum sample size may have affected model testing. Future research is required with larger samples to test the various characteristics of WHO-5.

## Conclusion

The Sinhala translation of WHO-5 well-being index has shown acceptable psychometric properties and can be used for assessing mental well-being in the Sinhala speaking community in Sri Lanka. Further testing of the measure with larger and diverse (including different ethnic/cultural) groups are indicated to test measurement invariance.

## Supplementary information

**Additional file 1.**

**Additional file 2.**

## Data Availability

The datasets supporting the conclusions of this article are included within the article (and its additional files).

## Abbreviations

ANOVA: Analysis of variance
AUC: Area Under the Curve
CFA: Confirmatory Factor Analysis
CFI: Comparative Fit Index
DFI: Differential Item Functioning
DSM: Diagnostic and Statistical Manual of Mental Disorders
ICC: Intraclass Correlation Coefficient
IRT: Item Response Theory
K10: Kessler Psychological Distress Scale
LMIC: Low-and-middle-income countries
MOH: Medical Officer of Health
NLR: Likelihood ration for a negative test
NPV: Negative Predictive Value
PHQ-9: Patient Health Questionnaire
PLR: Likelihood ratio for a positive test
PPV: Positive Predictive Value
RMSEA: Root Mean Square Error of Approximation
ROC: Receiver Operator Characteristics
SCID: Structured Clinical Interview for Diagnostic and Statistical Manual of Mental Disorders
SD: Standard Deviation
SEM: Structural Equation Model
SRMR: Standard Root Mean Square Residual
TLI: Tucker–Lewis Index
WHO-5: World Health Organization well-being index
WLSMV: Variance adjusted weighted least square estimator
