# Supplementary material for: Assessing mental well-being in a Sinhala speaking Sri Lankan population: validation of the WHO-5 well-being index
Source: Health Qual Life Outcomes. 2020 Sep 11;18:305. doi: 10.1186/s12955-020-01532-8 (PMC7488505; doi:10.1186/s12955-020-01532-8)
Supplement: Supplementary file 2 — Additional file 2. [file 12955_2020_1532_MOESM2_ESM.docx]

**Coding Guide for the database of Validation of WHO-5 Sinhala Version**

**Code**

A unique identification number given to all participants

Participants who have completed the paper-based questionnaire has a three-digit code starting with “R”. (Eg. R001)

Participants who have completed the online questionnaire has a three-digit code starting with “G” (Eg. G001)

**Age**

Participants’ age in years

**Sex**

“M” denotes Male

“F” denote Female

**District/Area**

The residence/home town of the participants

**Race**

The race of the participants are as follows.

1- Sinhala , 2- Sri Lankan Tamil, 3- Indian Tamil, 4- Muslim, 5- Burgher, 6- Malay, 7- Other

**Religion**

The religion of the participants are as follows

1-Buddhist, 2-Hindu, 3-Islam, 4- Roman Catholic, 5- Other Christian, 6-Other

**Occupational_Sector**

1-Government Employed, 2-Semi Government, 3-Private Sector employee, 4- Employer, 5-Self Employed, 6-Student, 7- Not employed

**Highest_Education**

1-Not attended school, 2-Pre-school, 3- Grade 1-13, 4-Degree and above (completed or continuing)

**WHO_Total**

Total score each respondent obtained for WHO-5 Sinhala version

**PHQ_Total**

Total score each respondent obtained for PHQ-9 screener

**K_Total**

Total score each respondent obtained for Kessler Psychological Distress Scale
